# Long-term evaluation of Elmelegy’s technique of local muscle transposition for the functional restoration of large upper or lower lip defects

**DOI:** 10.1186/s40902-024-00450-9

**Published:** 2024-12-18

**Authors:** Nader G. Elmelegy

**Affiliations:** Tanta University, Tanta, Egypt

**Keywords:** Lip reconstruction, Upper lip defect, Lower lip defect, Lip malignancy

## Abstract

**Background:**

Although more than 200 techniques have been reported for the reconstruction of the upper and lower lip defects since 1000 BC, none of them is ideal.

Local flaps may result in extra skin incisions and in some cases, the surgeon may be confronted with the lack of sufficient tissues for the reconstruction of large defects.

Several techniques have been described for near-total lip reconstruction. The two major available techniques are local flap reconstruction (Bernard–von Burrow–Webster technique) and free micro-vascular tissue transfer.

In this study, we are going to evaluate the functional results of using local muscles transposition-assisted dermal fat flap and muco-buccal flap in the treatment of large upper or lower lip defects.

**Materials and methods:**

This study was carried out on 128 patients, who presented to us with malignant tumors affecting the lips.

**Results:**

Lip defect sizes ranged from 4 to 6 cm in diameter. No flap failure was seen and desirable function and accepted esthetic results were obtained. The flap survival was 100%, and healing was eventful in all cases. No cases of microstomia were reported.

**Conclusion:**

The use of local muscle transposition-assisted dermal fat flap and muco-buccal flap technique, showed excellent results in regaining oral competence and lip mobility, and as much as possible, increased the aesthetic outcome.

**Level of evidence:**

IV therapeutic study.

**Supplementary Information:**

The online version contains supplementary material available at 10.1186/s40902-024-00450-9.

## Introduction

Since 1000 BC, over 200 methods for reconstructing upper and lower lip deformities have been described. However, none of them are appropriate for all types of errors [1]. Primary lip closure is the easiest method for modest tissue loss involving less than one-third of the lip and no significant microstomia [2].

The next phase in the repair procedure is to use local flaps, which have the advantages of good color matching and easy accessibility. On the other hand, local flaps can result in additional facial skin incisions. In certain cases, the surgeon may be unable to reconstruct severe deformities due to a shortage of adequate tissues [3].

For the reconstructive surgeon, a deformity that is one-third to two-thirds the length of the entire lip is a substantial challenge. The usage of a stepladder has been shown to yield excellent results. Two further procedures for repairing comparable malformations are trans-oral cross-lip flaps (Abbe and Estlander) and circum-oral advancement rotation flaps (Karapandzic and Gillies) [4].

When there are problems medial to the commissure, the Abbe flap might be used, whereas the Estlander flap includes the commissure in the design. The Abbe flap involves breaking the pedicle that crosses the oral stoma after 2 to 3 weeks [5]. Furthermore, sensory functions may take months to reconstitute since some flaps (Abbe, Estlander) have been denervated [6].

The Karapandzic flap is a rotation-advanced flap that gives excellent oral proficiency while keeping lip mobility. The disadvantage is that the lips’ circumference is lowered, which may lead to microstomia. Commissures have been rounded or deformed as well [7].

In the Bernard–von Burrow–Webster procedure, a cutaneous triangle excision advancement flap is employed. Four triangles of cheek skin are removed for the upper lip reconstruction, while three triangles of cheek skin are removed for the lower lip reconstruction [8]. Drooling is caused by this approach's adynamic reconstruction [4].

With good outcomes, free flaps have been employed in complete lip repair. The lower lip was completely restored using radial forearm and anterolateral thigh flaps. Free flaps’ oral function and cosmetic effects are limited because the texture of donor site tissue differs from that of facial skin [9, 10].

This study will investigate the long-term effects of using local muscle transfer in upper lip patients (bilateral upper half of the risorius muscle as a substitute for the excised bilateral upper half of the orbicularis orris, and reattachment of the levator labii superioris, levator labii superioris alaeque nasi, zygomaticus major, and zygomaticus minor to the dermis of the newly formed upper lip).

In terms of the lower lip (the bilateral lower half of the risorius muscle as a substitute for the excised bilateral lower half of the orbicularis orris, the remaining part of the depressor labii as a substitute for the mentalis, the depressor labii angularis as a substitute for the depressor labii and keeping attachment of platysma as a substitute for the depressor labii angularis).

## Materials and methods

A prospective clinical study was conducted at the author's private clinic and the plastic surgery department at Tanta University Hospitals in Egypt between February 2008 and July 2020. This study included 128 patients who came to see us with malignant tumors on their upper or lower lips. Local muscle transfer, dermal fat flap, and muco-buccal flap repair were used to achieve a satisfying functional outcome and as visually appealing lips as possible.

### Inclusion criteria

When they arrived at our department and a private clinic, all the patients had malignant lip tumors. The ensuing anomalies took up more than half of the damaged lip after the tumors were removed.

### Exclusion criteria

Patients with diabetes, connective tissue disease, and aspirin users were excluded from the trial. Patients with metastases, whether local or distant, were also removed from the study.

Name, age, sex, address, phone number, previous drug treatments, and any form of interference, whether surgical or lasers for treatment of the lip tumor, were all taken into consideration for all of the individuals who were chosen. Impact of the tumor on general health, lifestyle, physical, and social activity.

All patients who took part in the study gave their informed consent, which included information about the risks of the study, consent to clinical photography, and the potential of their data being published in medical journals.

### Preoperative markings: the upper lip (Fig. 1 )

**Fig. 1:**
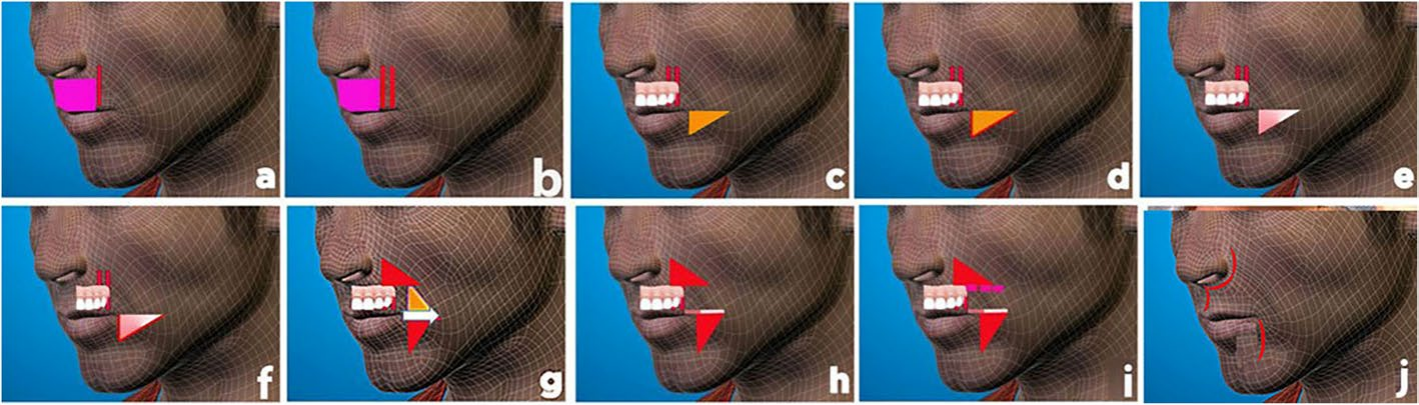
Operative steps in upper lip reconstruction. **a** A palpable tumor edge. **b** A margin of safety, c Triangular de-epithelialization. **d** Dermal fat flap. **e** The mucosal triangle of the buccal mucosa. **f** Muco-buccal flap. **g** Reducing the risorius in half. **h** The bottom section of the upper lip that has just formed. **l** preparing the newly formed upper part of the upper lip. **j** Closure of both sides

On the upper lip, the tumor's boundaries were evident (Fig. 1a). The safety margin was indicated around one centimeter from the tumor's margins in Fig. 1b. The proposed post-excisional defect was then measured. The layout of the flap was planned to use both the right and left cheeks. Starting at each angle of the mouth, the following approach was performed to mark half of the defect to be fixed on each cheek. A right-angle triangle was painted on the flesh of each cheek. The right angle of the triangle is near the angle of the mouth. Cupid’s bow on the upper lip was pointed in the same direction as the triangle's base, and the triangle was pointing down. The base of each triangle is half the length of the lip deformity that results (Fig. 1c). The same process was used to construct an opposing triangle on the mucosal side. Using the same procedures as in Fig. 1, a similar but somewhat larger opposing triangle was constructed (Fig. 1e). The procedure was repeated on the other cheek.

### For both upper and lower lip cases

The procedure was performed under general anesthetic in a sterile environment. Naso-tracheal intubation was utilized to avoid distortion of the tissues of interest and to give clear access to the mouth cavity. With 10% povidone-iodine, the entire face was sterilized. To cover the surrounding area, sterile towels were employed. The skin tumors were excised with an acceptable safety margin before initiating lip restoration, and histological confirmation of tumor-free margins was performed in all instances.

### Surgical technique: upper lip (Fig. 1)

On each side of the cheek, the previously mentioned skin triangle was de-epithelialized (Fig. 1c). As illustrated in Fig. 1, the dermal fat flap was then lifted at the same triangle, keeping its base in the direction of Cupid’s bow on the upper lip and supplying random blood supply to the flap (Fig. 1d). After that, we went over to the oral cavity. The somewhat larger previously indicated triangle was elevated on the mucosal side, opposite the previous one, retaining its base in the direction of Cupid’s bow as well (Fig. 1e, f). Reflecting the cutaneous fat flap externally upwards and the muco-buccal flap internally upwards, the muscles up to the risorius muscle were exposed (Fig. 1g). As indicated in Fig. 1, the risorius muscle was divided starting at the angle of the mouth and extended laterally till the end of each triangle's base (Fig. 1g). The cutaneous fat flap was then used to wrap the upper half of the spitted risorius muscle. The mucosal flap was sutured over the dermal fat flap to obtain an appropriate aesthetic upper lip contour, as shown in Fig. 1h. As a result, the bottom half of the newly styled upper lip was completed. Another triangle of skin and subcutaneous tissue was taken from the upper half of the newly created lip at the nasolabial fold (Fig. 1g). The entire section, including the risorius muscle, was relocated medially after the base of the triangle was surgically removed (Fig. 1h). The operation was repeated on the other cheek. To cover the lip defect, the two newly formed segments were advanced medially and sutured together, mucosa to mucosa and the risorius of one side to the risorius of the other side, to produce a sphincter as a replacement for the excised orbicularis orris muscle (Fig. 1i). On both sides at the upper part of the newly formed lip, the remaining parts of the levator labii superioris, levator labii superioris alaeque nasi, zygomaticus minor, and zygomaticus major are re-sutured to the dermis of the upper part of the newly formed lip. The freshly created upper lip skin, as well as the consequent triangles, were then closed, resulting in the construction of a new upper lip, as seen in Fig. 1j. Following that, the dressing was put on. All patients were given a single dose of a broad-spectrum antibiotic for 3 days after surgery.

### Preoperative markings: lower lip (Fig. 2)

**Fig. 2:**
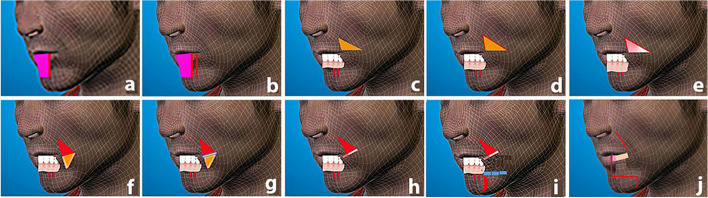
Marking and operative steps of lower lip reconstruction. **a** Palpable edge of the tumor. **b** Safety margin. **c** De-epithelialized triangle. **d** Dermal fat flap. **e** Muco-buccal flap. **f** Reflecting both flaps downwards. g Cutting through the risorius. **h** Formation of the upper part of the newly formed lip. **i** Mento-labial incision and subcutaneous muscle cutting. **j** Left lateral view after closure

The edge of the tumor at the lower lip is seen in Fig. 2a. The one-centimeter safety zone surrounding the tumor’s edge is shown in Fig. 2b. The potential post-excision fault was then assessed. During the flap plan, each cheek limb is half the length of the horizontal lip deficiency. Starting at each mouth angle, the following process was performed to mark half of the defect to be fixed on each cheek. A right-angled triangle was drawn on the flesh of each cheek. The right angle of the triangle is stated near the mouth. The base of the triangle was facing upwards, in the same direction as Cupid’s lower lip bow, as shown in Fig. 2c, d. The base length of each triangle is equal to half of the resulting lip deformity. A comparable but somewhat larger opposite triangle was created on the mucosal side, using the same approaches as in Fig. 2e. The operation was repeated on the other cheek.

### Surgical technique: lower lip (Fig. 2)

The edge of the tumor at the lower lip is seen in Fig. 2a. The one-centimeter safety zone surrounding the tumor's edge is shown in Fig. 2b. On each side of the cheek, the previously marked skin triangle was de-epithelialized (Fig. 2c). The dermal fat flap was then elevated in the same triangle, preserving its base in the direction of Cupid’s bow and supplying the flap with random blood supply (Fig. 2d). Following that, we'll look at the oral cavity. The somewhat larger, previously sketched triangle was elevated on the mucosal side opposite the prior one, keeping its base in the Cupid’s bow orientation (Fig. 2e). Internally reflecting the muco-buccal flap downwards and externally reflecting the dermal fat flap downwards helped expose the muscles up to the risorius muscle in Fig. 2f. In Fig. 2, the risorius muscle was divided starting at the angle of the mouth and extending laterally to the base of each triangle (Fig. 2g). The cutaneous fat flap was then used to repair the lower section of the divided risorius muscle. The mucosal flap was sutured over the dermal fat flap to obtain an appropriate aesthetic lip contour (Fig. 2h). The skin and muscles at the level of the lower border of the jaw were separated through an incision made at the mento-labial sulcus at the lower region of the newly formed lip. The mucous membrane and remaining depressor labii and depressor labii angularis sections were severed at this level, but the skin above it was left intact (Fig. 2). The platysma muscle, which is connected to the mouth's angle, is also retained. The newly formed lip segments on both sides were advanced medially and sutured together. On one side, the lower half of the risorius links to the lower half of the risorius on the other side, developing the oral sphincter's lowest portion (as a substitute for the excised orbicularis orris sphincter). The remaining part of the depressor labii was sutured to the remaining origin of mentalis, and the remaining part of the depressor labii angularis was sutured to the remaining part of the depressor labii angularis was sutured to the remaining part of the depressor labii Finally, the skin and mucus membrane triangles were closed, and sutures were placed on both sides of the lip skin to close the midline and mento-labial sulcus. As a result, as shown in Fig. 2, a new lower lip has developed (Fig. 2j). Following that, the dressing was put on. All patients were given a single dose of a broad-spectrum antibiotic for 3 days after surgery.

### Post-operative care and follow-up

The patients were discharged on the second postoperative day and returned for stitches removal on the seventh postoperative day. Patients were followed up for 2 weeks, 1 month, 3 months, and 6 months or more following discharge from the hospital. After at least 6 months, the following data were collected: The preoperative photography session and the photography session during the last follow-up appointment were both deemed postoperative photographic results if they were done 6 months or more following surgery. Data on the functional and aesthetic outcomes were gathered. Data on unfavorable cosmetic outcomes, Scar issues, oral competence, drooling, and speech difficulties have all been reported. Symptomatic pain, difficulty performing daily tasks, difficulty sleeping in a comfortable position, and a deterioration in the quality of life were also observed. Patient satisfaction was assessed using patient-reported outcome measures (PROMs), which were questionnaires that assessed the patient’s pleasure with the following parameters: functional and aesthetic shape, lifestyle, relatives’ opinions, and overall contentment. The information gathered was categorized and tallied. The patients were asked to rank their level of satisfaction on a scale of 1 (poor), 2 (fair), 3 (good), and 4 (outstanding), with 1 being the lowest and 4 being the highest. Clinical satisfaction was measured using preoperative and late postoperative photographs by three plastic surgeons who were not involved with the study. The results of the preoperative and last follow-up photos were rated as excellent, good, fair, and poor on a scale of 1 to 4.

## Results

A total of 92 (71.9%) males and 36 (28.1%) females were among the 128 patients. Squamous cell carcinoma was found in all the patients, with 23 (16.9%) having upper lip cancer and 105 (82.1%) having lower lip cancer. The patients’ ages ranged from 31 to 68, with a median of 47 years. The defect occupied more than two-thirds of the lip (4–5 cm) in 13 (56.5%) of the 23 cases with upper lip defect and more than four-fifths of the lip (5–6 cm) in 10 (43.5%) patients. A total of 105 cases of lower lip deformity were examined, the defect occupied more than two-thirds of the lip (4–5 cm) in 84 (80%) of the instances, and more than four-fifths of the lip (5–6 cm) in 21 (20%) of the patients. A bigger deformity, extending beyond the oral commissures, was found in 9 (8.6%) of the patients. The cutaneous fat flap, local muscle transfer, and muco-buccal flap were used on all the patients. There were no prophylactic neck dissections performed. On the second surgical day, oral feeding began.

Seventy-two patients (56.25%) obtained complete oral competency out of a total of 128. After surgery, 56 patients experienced solely postoperative drooling for fluids, with 7 (30.4%) upper lip instances and 49 (46.7%) lower lip cases. Drooling severity was recorded using a questionnaire designed to evaluate a patient's drooling severity throughout daily activities such as standing, sitting, lying down, eating, and talking. There were 40 mild cases (71.4%) 12 intermediate cases (12.4%), 12 moderate cases (21.4%), and 4 severe cases (7.2%). After 3 to 6 months, everything had entirely improved.

All of the patients had a successful one-stage near-total lower lip restoration, with no requirement for a second stage for flap division and inset. The follow-up period ranged from 1 to 10 years, with eight patients missing out. Lip defects ranged in size from 4 to 6 cm in diameter. There was no flap failure, and the function and aesthetic results were satisfactory. The flap survived 100% of the time, and healing was uneventful in every case. There were no cases of microstomia reported. However, macrostomia affected 8 (6.3%) of the patients. They were rectified under local anesthesia, and the recovery process went smoothly. All patients were discharged without a feeding tube on the second postoperative day and were required to do daily oral hygiene.

Three plastic surgeons who were not involved in the study conducted a clinical evaluation using a four-point scale. Six (26.1%) patients had excellent outcomes, 12 (52.2%) patients had good results, 5 (21.7%) patients had fair results, and no patients had bad results. Excellent results were reported in 15 (14.3%) instances, acceptable results in 80 (76.2%), and fair results in 10 (9.5%) and no poor results were documented in lower lip cases.

**Fig. 3:**
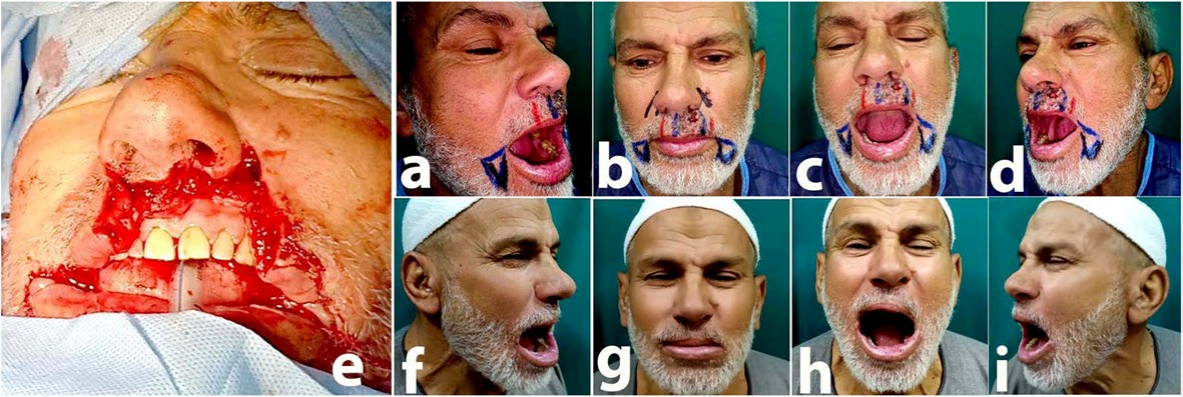
**a**–**d** Preoperative views: (**a**) right lateral (**b**) and post with a closed mouth, (**c**) with open mouth, (**d**) left lateral, and (**e**) intraoperative view after tumor excision. **f**–**i** Two years postoperative views. **a** Right lateral, (**b**) post with a closed mouth, (**c**) with open mouth, and d left lateral

**Fig. 4:**
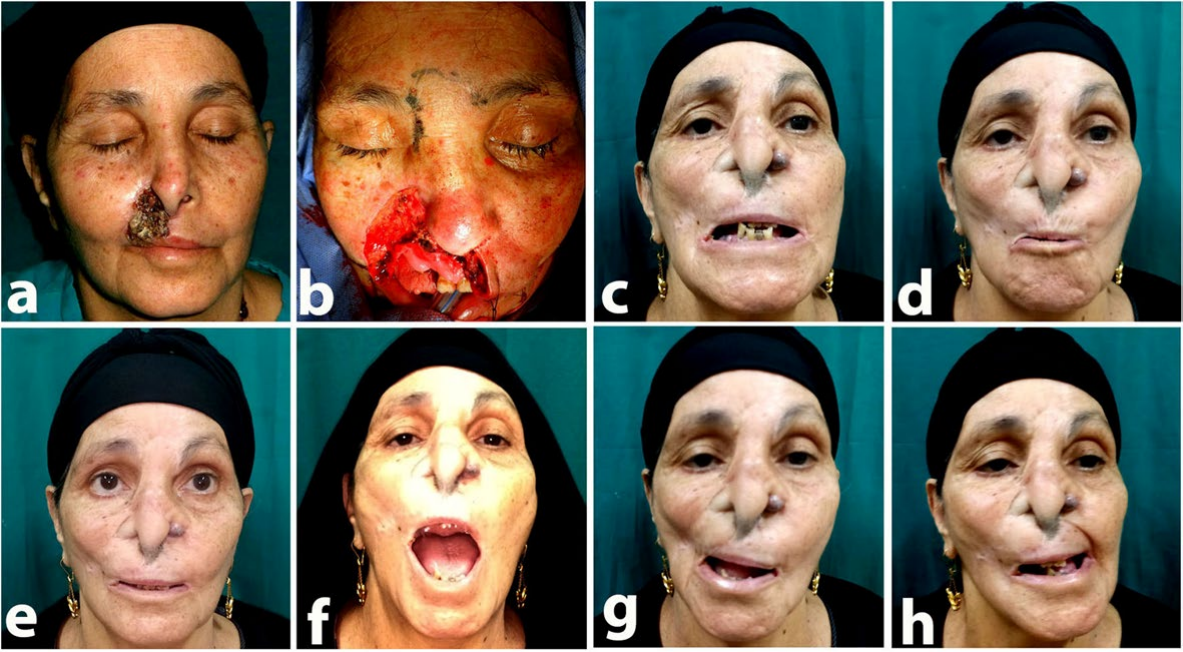
**a** Preoperative frontal view showing upper lip epithelioma. **b** The intraoperative view showing upper lip defect, 6 years post-operative views showing our technique for upper lip and forehead flap for nasal reconstruction., **c** showing her teeth, **d** whistling, **e** closed mouth, **f** open mouth, **g** working RT upper lip elevator, **h** working LT upper lip elevators

**Fig. 5:**
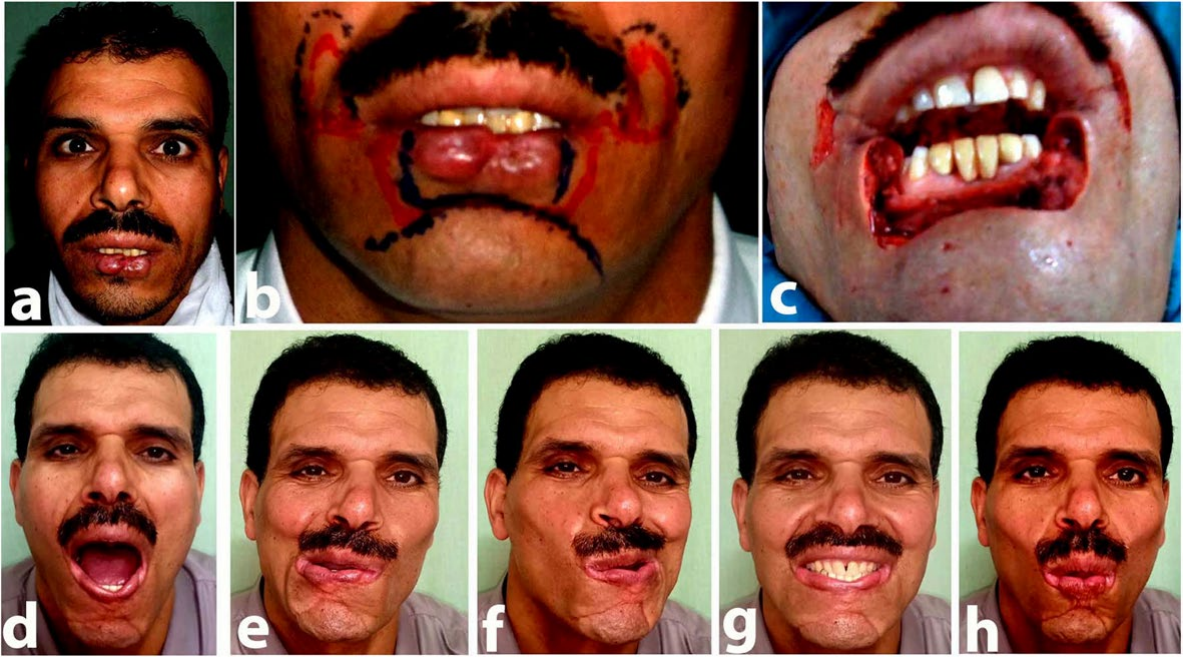
**a** Preoperative frontal view, **b** preoperative nearby frontal view showing lower lip epithelioma, **c** the intraoperative frontal view showing the lower lip defect. Nineyears post-operative views, **d** open mouth, working. **e** RT lip elevators, **f** working LT lip elevators, **g** showing his teeth, **h** whistling

**Fig. 6:**
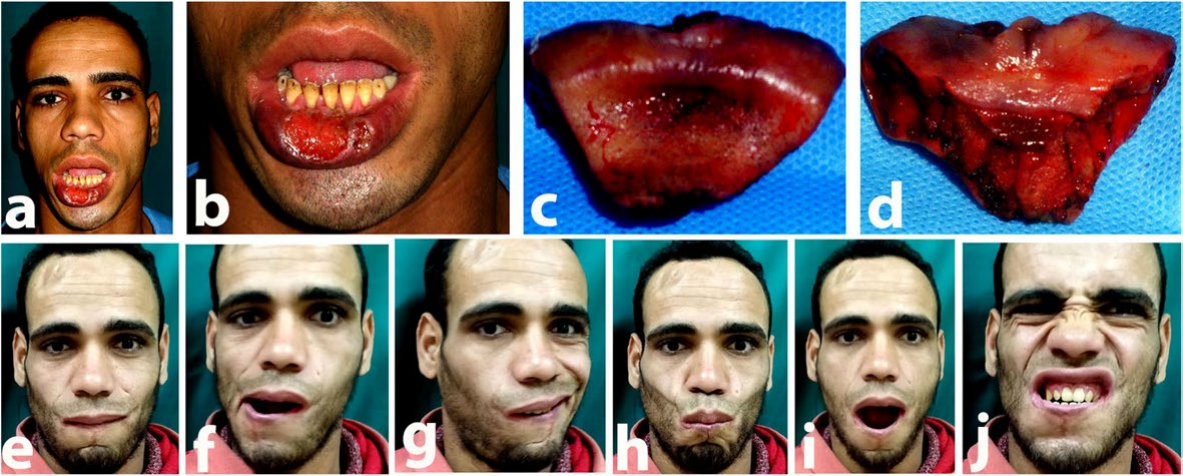
**a** Preoperative frontal view, **b** preoperative nearby frontal view showing lower lip epithelioma, **c** intraoperative frontal view of the excised part of the upper lip, **d** intraoperative back view of the excised upper lip defect. Five years post-operative views, **e** closed mouth, **f** working RT upper lip elevator, **g** working LT upper lip elevators, **h** whistling, **i** open mouth, **j** showing his teeth

PROMs, questionnaires that measure the patients' satisfaction with the following parameters: aesthetic shape, lifestyle, relatives' comments, and general satisfaction, were used to assess patient satisfaction. Excellent results were recorded in 8 (24.8%) instances, good results in 11 (47.8%), fair results in 4 (17.4%), and no poor results were reported in the upper lip cases. In the lower lip cases, great results were seen in 20 (19%) patients, good results in 78 (74.3%) patients, acceptable results in 7 (6.7%) patients, and no poor results (Table 1, Figs. 3, 4, 5, and 6).

**Table 1:** Descriptive-analytic data for age, gender, defect size, microstomia or macrostomia, oral competence, drooling, patient satisfaction, and clinical satisfaction

Variable	Upper lip patients (23)	Percentage %	Lower lip patients (105)	Percentage %
Age				
40–50 years	3	13.1%	14	13.3%
50–60 years	7	30.4%	26	24.8%
Above 60 years	13	56.5%	65	61.9%
Sex				
Male	10	43.5%	82	78.1%
Female	13	56.5%	23	21.9%
Size of the defect:				
4–5 cm	13	56.5%	84	80%
5–6 cm	10	43.5%	21	20%
Microstomia	0	0%	0	0%
Macrostomia	2	8.7%	6	5.7%
Oral competence	14	60.9%	58	55.2%
Drooling	9	(39.1%)	47	(44.8%)
Mild	5	21.7%	35	33.3%
Moderate	3	13.1%	9	8.6%
Severe	1	4.3%	3	2.9%
Patient satisfaction:				
Excellent (4)	8	34.8%	20	19.1%
Good (3)	11	47.8%	78	74.3%
Fair (2)	4	17.4%	7	6.6%
Poor (1)	0	0%	0	0%
Clinical satisfaction:				
Excellent (4)	6	26.1%	15	14.3%
Good (3)	12	52.2%	80	76.2%
Fair (2)	5	21.7%	10	9.5%
Poor (1)	0	0%	0	0%

## Discussion

During lip repair, surgeons must consider the defect’s characteristics, such as residual tissues after tumor excision and skin laxity. Surgeons must keep up with the current breakthroughs in lip restoration to present their patients with the best possibilities. Lip defects that cover more than a third of the lip require specialized treatment.

For lower-lip reconstruction, Ebrahimi et al. [7] used four distinct types of local flaps: Karapandzic, Estlander, stepladder, and reversed-Abbe. They discovered that the reversed Abbe flap and the stepladder flap produced the same functional and aesthetic results. They preferred the single-stage stepladder flap over the two-stage reversed-Abbe flap, nevertheless. Patients receiving the Estlander flap had no functional issues, however, they did have lip asymmetry, according to the researchers. In patients with defects occupying more than half of the lip, they suggested using a bilateral reversed Abbe flap instead of the Karapandzic flap. We employed local muscle transfer to aid dermal fat flap and muco-buccal flap in all individuals with extensive defects occupying up to four-fifths of the upper or lower lips in our research. The products were both utilitarian and aesthetically pleasing.

The Abbe flap is a two-stage surgery for abnormalities in the commissure’s medial region, whereas the Estlander flap includes the commissure in the design. The main disadvantages of the Abbe flap are that it is a two-stage surgery and that there is a risk of the flap being completely lost [11]. Another disadvantage of Abbe or Estlander flaps is that they are denervated, which means that sensations may not return for several months. Because it creates scars that extend to the free vermilion edge and can result in a trapdoor deformity, the Abbe flap or its variants may not provide adequate results [12]. It was a phased treatment with good results in our technique, and no flap was lost. The motor and sensory supply were maintained in all cases, and there was no trap-door deformity.

The submental island flap is a loco-regional flap that can aid in lip deformity restoration. It has the benefits of being a dependable flap with axial-pattern blood supply, great color match, acceptable tissue thickness, and ease of harvest [13]. However, because it is merely a skin flap with no muscles, it is an adynamic flap. All lip functions were restored using our procedure, which included the three layers of the lip: skin, muscles, and mucus membrane.

Hanasono and Langstein [14] advocated the Karapandzic approach as the first choice for bigger lip lesions that occupy more than one-half of the lip and have sufficient lip tissue, and the Estlander technique as the second choice for lip reconstruction. In patients with greater lesions, Aldelaimi et al. [15] used the extended Karapandzic flap approach as an alternative to micro-vascular free flaps, with good results. The generated round commissures are the fundamental shortcomings of Karapandzic and Estlander’s approach. The lip’s muscle activities in speech, sphincteric action, kissing, whistling, and exposing teeth were all adequately documented using our technique. Patients also had excellent oral commissures following surgery.

The Bernard–von Burow–Webster procedure is a cutaneous triangle excision advancement flap. The upper lip is reconstructed by removing four triangles of cheek skin, while the lower lip is reconstructed by removing three triangles. Although they were originally described as full-thickness excisions, they are usually only conducted on the skin [16]. Unfortunately, this method is ineffective and causes drooling, trouble kissing, and whistling. Because the reconstruction is only cutaneous, any mucosal abnormalities must be corrected individually. Because there is less chance of developing postoperative oral incompetence, this approach is probably better suited for upper lip reconstruction [5]. Local muscle transfers because of medial muscle advancement produced outstanding functional effects in our investigation.

Microvascular free tissue transfers such as the radial forearm flap or the anterolateral thigh flap are sometimes required for the restoration of large lip deformities. It enables the repair of significant lip abnormalities in a single stage, encompassing afflicted parts of the cheek and/or chin if present. Anterolateral thigh flaps and free radial forearm flaps have been proven to be reliable procedures [9, 17–19].

Our procedure was dynamic and had the same principles performed in Elmelegy [20] preserving oral competency, maximum oral aperture, and lip mobility while improving the cosmetic outcome as much as feasible. In upper lip cases, the remaining sections of the levator labii superioris, levator labii superioris alaeque nasi, zygomaticus minor, and zygomaticus major were re-sutured to the dermis of the upper part of the newly created lip on both sides at the upper part of the newly formed lip. The top half of one side's risorius was sutured to the upper half of the opposite side’s risorius to produce a sphincter in place of the removed orbicularis orris muscle.

The lower half of one risorius was sutured to the lower section of the risorius on the opposite side, forming the lower part of the oral sphincter in lower lip cases (as a substitute for the orbicularis orris sphincter). The remaining part of the depressor labii was sutured to the remaining origin of the mentalis at the lower part of the newly formed lower lip, the remaining part of the depressor labii angularis was sutured to the remaining origin of the depressor labii, and the platysma was kept inserted into the mouth angle.

Our method was a one-step process that produced acceptable functional and aesthetic benefits. Patient satisfaction was excellent in 8 (24.8%) instances, good in 11 (47.8%), and fair in 4 (17.4%) cases in upper lip cases, with no poor results reported. Lower lip instances yielded outstanding results in 20 (19%) cases, good results in 78 (74.3%) cases, acceptable results in 7 (6.7%) cases, and no poor results. In terms of clinical satisfaction, 6 (26.1%) patients had excellent outcomes, 12 (52.2%) patients had good results, 5 (21.7%) patients had fair results, and no patients had bad results. In lower lip cases, 15 (14.3%) patients had great outcomes, 80 (76.2%) patients had good results, and 10 (9.5%) patients had fair results.

## Conclusion

Local muscle transfer can provide highly promising results from a functional standpoint in terms of reconstruction.

The long-term results of employing local muscle transfer-assisted dermal fat flap and muco-buccal flap procedures in restoring oral competence, maximum oral aperture, and lip movement were outstanding, and the cosmetic outcome was improved as much as feasible.

## Supplementary Information

Supplementary Material 1.

## Data Availability

No datasets were generated or analysed during the current study.

## Abbreviations

PROMs: Patient-reported outcome measures

## References

[1] Dediol E, Čvrljević I, Dobranić M, Uglešić V (2018) Extended Karapandzic flap technique for reconstruction of lower lip and chin defect. J Oral Maxillofac Surg 76(1):213–220 28697350 10.1016/j.joms.2017.06.015

[2] Lubek J, Ord R (2013) Lip reconstruction. Oral Maxillofac Surg Clin North Am 25:203–214 23510600 10.1016/j.coms.2013.01.001

[3] Pepper J, Baker S (2013) Local flaps: cheek and lip reconstruction. JAMA Facial Plast Surg 15(5):374–382 24051684 10.1001/jamafacial.2013.1608

[4] Milomir N, Stefano S, Karen F, Marina N (2010) Lower lip reconstruction using a functioning gracilis muscle free flap. Semin Plast Surg 24(2):212–218 22550441 10.1055/s-0030-1255338 PMC3324240

[5] Ashish K, Premlatha MS, Rohan S, Shashank R, Ram M, Harsh K (2014) The versatility of abbe-estlander flap in lip reconstruction – a prospective clinical study. J Clin Diagn Res 8(10):NC18–NC21 25478393 10.7860/JCDR/2014/10661.5057 PMC4253211

[6] Uglesic V, Amin K, Dediol E, Kosutic D (2019) Combined Karapandzic-Abbé/Estlander/Stein flap for subtotal and total lower lip reconstruction. J Plast Reconstr Aesthet Surg 72(3):484–490 30660466 10.1016/j.bjps.2018.11.005

[7] Ebrahimi A, Maghsoudnia G, Arshadi A (2011) Prospective comparative study of lower lip defects reconstruction with different local flaps. J Craniofac Surg 22:2255–2259 22075831 10.1097/SCS.0b013e318232786d

[8] Brinca A, Andrade P, Vieira R, Figueiredo A (2011) Karapandzic flap and Bernard-Burrow-Webster flap for reconstruction of the lower lip. AnBras Dermatol 86(4 Suppl 1):S156–S159 10.1590/s0365-05962011000700041 22068799

[9] Daya M, Nair V (2009) Free radial forearm flap lip reconstruction: a clinical series and case reports of technical refinements. Ann Plast Surg 62:361–367 19325337 10.1097/SAP.0b013e31818b4515

[10] Ramesh S, Mohd A, Normala H (2012) Lip and oral commissure reconstruction with the radial forearm flap. Natl J Maxillofac Surg 3(1):21–24 23251053 10.4103/0975-5950.102144 PMC3513804

[11] Okamoto T (2020) Reconstruction of an upper lip and intraoral defect following resection of an upper lip melanoma using a lower lip musculomucosal flap combined with a tongue flap. J Surg Case Rep 2020(4):rjaa072 32280446 10.1093/jscr/rjaa072 PMC7136836

[12] Genc S, Ugur S, Arslan I, Tuhanioglu B, Demir A, Selcuk A (2012) Lower lip reconstruction with Abbe-Estlander flap modification: preserving the same side vascular pedicle. Eur Arch Otorhinolaryngol 269:2593–2594 22639198 10.1007/s00405-012-2052-1

[13] Guo Y, Mao C (2016) The use of submental island flap for total lower lip reconstruction: a case report. Facial Plast Surg 32(2):238–239 27097147 10.1055/s-0036-1571810

[14] Hanasono M, Langstein H (2011) Extended Karapandzic flaps for near-total and total lower lip defects. Plast Reconstr Surg 127:1199–1205 21364422 10.1097/PRS.0b013e318205f3ce

[15] Aldelaimi TN, Khalil AA (2014) Lip reconstruction using Karapandzic flap. J Craniofac Surg 25(2):e136–e138. 10.1097/SCS.0000000000000462. PMID: 24481165 24481165 10.1097/SCS.0000000000000462

[16] Inês C, Leonor R, Ana R, Ricardo V, Américo F (2015) Lower lip reconstruction with nasolabial flap - going back to basics. AnBras Dermatol 90(3 Suppl 1):206–208 10.1590/abd1806-4841.20153714 PMC4540552 26312718

[17] Sasidaran R, Zain M, Basiron N (2012) Lip and oral commissure reconstruction with the radial forearm flap. Natl J Maxillofac Surg 3(1):21–24 23251053 10.4103/0975-5950.102144 PMC3513804

[18] Tan O, Kuduban S, Algan S, Cinal H, Barin E (2013) Total lower lip reconstruction using free neurotendino-fasciocutaneous anterolateral thigh composite flap: a case report. J Reconstr Microsurg 29(7):487–490 23670442 10.1055/s-0033-1343833

[19] Shinohara H, Iwasawa M, Kitazawa T, Kushima H (2000) Functional lip reconstruction with a radial forearm free flap combined with a masseter muscle transfer after wide total excision of the chin. Ann Plast Surg 45(1):71–73. 10.1097/00000637-200045010-00014. PMID: 10917103 10917103 10.1097/00000637-200045010-00014

[20] Elmelegy N, El Sakka D (2017) One stage aesthetic and functional reconstruction of major lower lip defects. Ann Plast Surg 78(4):417–420 27984219 10.1097/SAP.0000000000000918

